# Beta-blockers and mechanical dyssynchrony in heart failure assessed by radionuclide ventriculography

**DOI:** 10.1007/s12350-022-03142-x

**Published:** 2022-11-23

**Authors:** K. A. Jones, C. A. Paterson, S. Ray, D. W. Motherwell, D. J. Hamilton, A. D. Small, W. Martin, N. E. R. Goodfield

**Affiliations:** 1 Department of Nuclear Cardiology, Glasgow Royal Infirmary, Glasgow, UK; 2 School of Physics and Astronomy, University of Glasgow, Glasgow, UK; 3 School of Mathematics and Statistics, University of Glasgow, Glasgow , UK; 4 School of Medicine, Dentistry and Nursing, University of Glasgow, Glasgow, UK

**Keywords:** Cardiac dyssynchrony, beta-blockers, heart failure, RNVG phase, approximate entropy, sample entropy

## Abstract

**Background:**

Radionuclide ventriculography (RNVG) can be used to quantify mechanical dyssynchrony and may be a valuable adjunct in the assessment of heart failure with reduced ejection fraction (HFrEF). The study aims to investigate the effect of beta-blockers on mechanical dyssynchrony using novel RNVG phase parameters.

**Methods:**

A retrospective study was carried out in a group of 98 patients with HFrEF. LVEF and dyssynchrony were assessed pre and post beta-blockade. Dyssynchrony was assessed using synchrony, entropy, phase standard deviation, approximate entropy, and sample entropy from planar RNVG phase images. Subgroups split by ischemic etiology were also investigated.

**Results:**

An improvement in dyssynchrony and LVEF was measured six months post beta-blockade for both ischemic and non-ischemic groups.

**Conclusions:**

A significant improvement in dyssynchrony and LVEF was measured post beta-blockade using novel measures of dyssynchrony.

## Introduction

Heart failure affects approximately 1%-2% of the adult population in developed countries, increasing to over 10% for those > 70 years.^1-4^ Various treatments are available which aim to improve symptoms, morbidity, and mortality. However, identifying the underlying cause is crucial to determine the most appropriate treatment. This research focuses on non-valvular heart failure with reduced ejection fraction (HFrEF). In HFrEF, LVEF is known to be a good predictor of outcome, and is included in the decision criteria for many HFrEF treatments.^5^

There is some interest in measures of left ventricular dyssynchrony for patients with HFrEF. For example, there have been many studies published investigating imaging parameters as predictors to cardiac resynchronization therapy response, with varying degrees of success as summarized in the review by Hawkins et al.^6^ Despite promising results in single-center studies,^7,8^ the results have not been reproduced in larger multi-center trials,^9^ leaving many unanswered questions in this area.

Beta-blockade therapy is well established and currently recommended by the ESC guidelines as first-line treatment for patients in symptomatic HFrEF.^5^ Several large-scale clinical trials have demonstrated the benefits of beta-adrenoceptor blockers for heart failure patients, with a reduction in morbidity and mortality.^10,11^ It is known that post beta-blockade therapy, patients show significantly improved LVEF,^12^ but the effect of beta-blockade on cardiac dyssynchrony has not been widely investigated. There are several published studies that investigate the use of echo-derived dyssynchrony parameters, such as septal to lateral wall delay, for heart failure patients.^13-15^ However, the authors are not aware of any published studies investigating the effect of beta-blockade on dyssynchrony measured from radionuclide ventriculography (RNVG) imaging.

### Dyssynchrony measures from RNVG phase analysis

RNVG phase parameters offer an alternative index for the quantification of ventricular dyssynchrony and may be a valuable adjunct in the assessment of patients with heart failure. Various measures to quantify dyssynchrony, including synchrony, entropy, approximate entropy (*ApEn*), and standard deviation of the phase histogram (phase SD), can be used to provide additional information from planar RNVG images with high reproducibility.^16-23^ Sample entropy (*SampEn*)^24^ is novel for this application and has not been previously investigated.

This work aims to investigate the effect of beta-blocker therapy on dyssynchrony, as assessed by planar RNVG phase images, for symptomatic HFrEF. Subgroups split by ischemic etiology will also be compared to determine if there is a difference in response.

## Method

### Study outline

A retrospective study was carried out for 98 heart failure patients who attended the department in 2005-2006. The inclusion criteria are defined in Table 1. All patients who were included had evidence of left ventricular systolic dysfunction, NYHA class II-IV, and were stabilized on standard HF treatment. Patients who had recent intervention, including CABG, PCI, CRT, and RV pacing, were excluded to ensure that any change in function would be secondary to beta-blocker and not intervention related. Those with atrial fibrillation or severe valve disease were excluded as these conditions can make the assessment of LV systolic function less reliable. None of the patients who were included had any intervention between the baseline and follow-up RNVG. No other heart failure medications were changed during this period.

All of the patients included had a planar RNVG and Thallium-201 myocardial perfusion (MPI) scan pre titration of beta-blocker and a repeat RNVG six months post beta-blocker. Patients were initially given 1.25 mg of Bisoprolol, with the dose increasing stepwise to 2.5 mg, 5 mg, 7.5 mg, and 10 mg at intervals of two weeks. Before each step increase, patients underwent clinical review. Each patient continued on their maximum tolerated dose of Bisoprolol. Patients who did not tolerate the prescribed beta-blockers and those who did not attend the second RNVG scan were excluded from this study. Of the 12 patients who were excluded, 8 patients did not tolerate beta-blocker, 3 patients did not attend for the second RNVG for unknown reasons, and 1 patient died before the second RNVG. After the exclusion criteria were applied, there were 86 patients remaining.

**Table 1:** Study inclusion and exclusion criteria

*Inclusion criteria*	
Left ventricular systolic dysfunction as assessed by echo or planar RNVG (LVEF < 40% )	
Chronic stable HF symptoms (NYHA class II-IV)	
Stabilized on standard HF treatment (without beta-blocker)	
Clinically stable and free from all cause admission for 1 month	
*Exclusion criteria*	
Use of beta-blockers in the last 6 months	
Asthma or COPD with significant reversibility on PFTs	
Atrial fibrillation	
PCI within 3 months	
CABG within 6 months	
MI within 1 year	
Resting HR < 60 bpm	
Sitting systolic blood pressure < 85 mmHg	
Severe valve disease	

*HF*, heart failure; *NYHA*, New York Heart Association; *COPD*, chronic obstructive pulmonary disease; *PFTs*, pulmonary function tests; *PCI*, percutaneous coronary intervention; *CABG*, coronary artery bypass graft; *MI*, myocardial infarction

The patients were grouped depending on whether or not they had heart failure of ischemic etiology. Of this patient cohort, 54 were ischemic, and 32 were non-ischemic. A patient was categorized in the ischemic heart failure group if at least one of the following criteria was met:

(i) A stenosis of more than 50% in at least one of the three major coronary arteries as assessed by coronary angiogram (where available)
(ii) Previous MI or PCI
(iii) Positive Thallium-201 MPI (defined by two experienced reporters).

### Data acquisition and processing

All patients underwent planar RNVG imaging before and six months after beta-blockade. In-vivo labeling was performed using intravenous administration of pyrophosphate 20 minutes prior to injection of technetium-99m pertechnetate. The administered dose for each scan was 600 MBq (16.2 mCi). The gamma camera was positioned to achieve the best septal separation between the left and right ventricles. Imaging was acquired on an Optima gamma camera (GE Healthcare, Waukesha, WI) using list mode acquisition and processed using MAPS Link Medical 10000 software. The raw data were reconstructed into a 24 frame 64 × 64 matrix with the exclusion of heartbeats 10% greater than the mean inter-beat (R–R) interval. All data were reviewed to check heart rate, gating, image quality, and ensure adequate septal separation. The acquisition angle was recorded to ensure the same angle was used for the repeat scan.

The pre and post therapy images were anonymized and randomized before analysis. LVEF was calculated from the gated images by an experienced operator and reviewed by a second operator. To assess intra-operator variability each of the anonymized images were analyzed twice by the same operator, without reference to their first ROI. A manual single region of interest method was used following the standard clinical protocol at the time of the study. The single region of interest technique systematically underestimates the ejection fraction, but has good reproducibility. The locally established normal range for LVEF by this method is > 40% and the inter-observer variability is 3.1%.^25^

### Phase analysis

**Figure 1:**
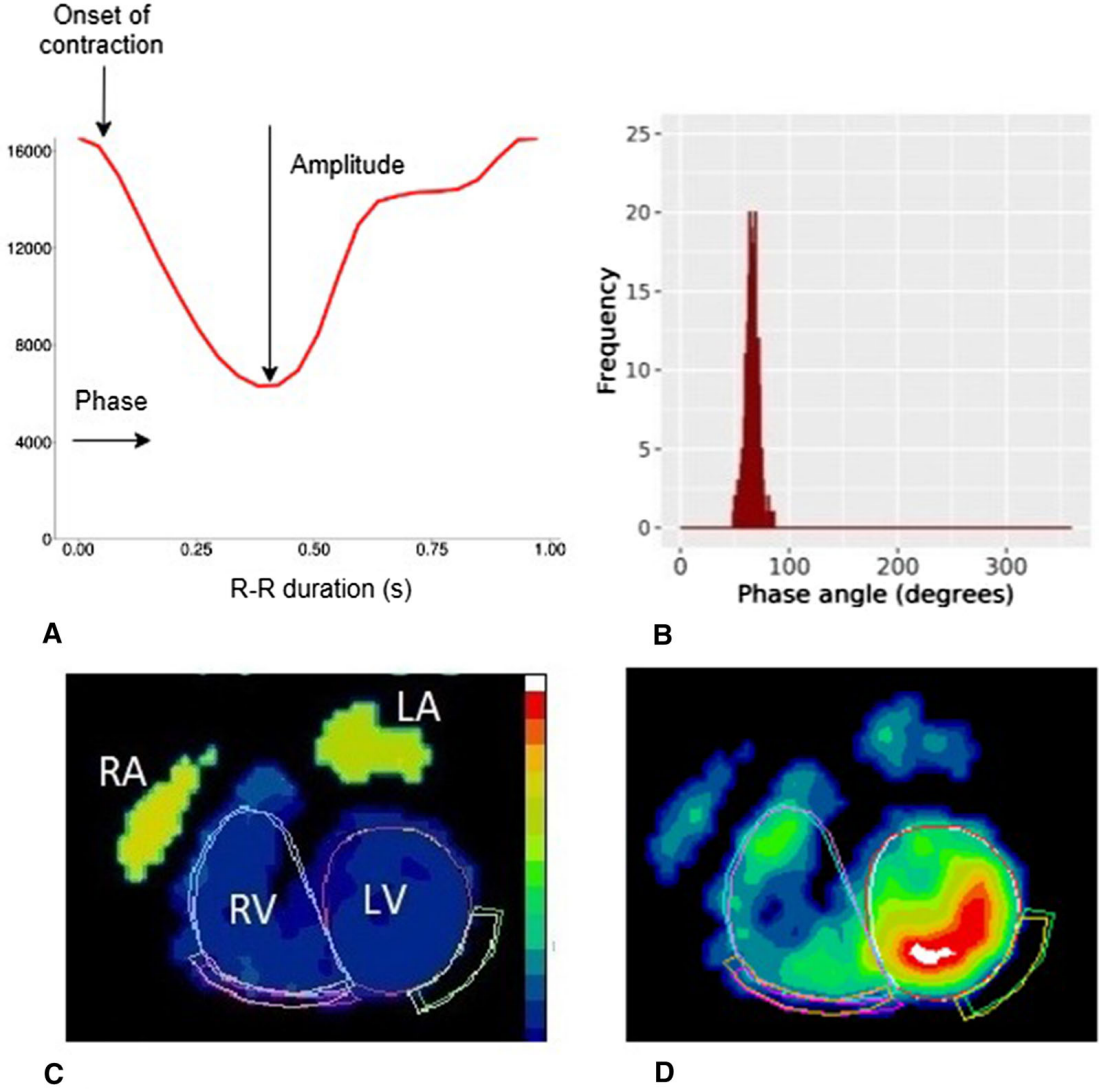
Example from a patient with synchronous contraction showing (**A**) the time–activity curve for the LV, (**B**) the phase histogram, (**C**) a phase image representing the timing of contraction with similar phase values within the ventricles, and (**D**) the amplitude image showing the maximum change in counts

Phase images were created using a first-order harmonic fit of the time–activity curve for each pixel, representing the timing of ventricular contraction relative to the R wave of the ECG.^26,27^ The phase angle defines the point in the time–activity curve where the Fourier function reaches its peak, representing the onset of contraction. An example of a phase and amplitude image with the associated time–activity curve and phase histogram for a patient with normal ventricular contraction is shown in Figure 1. The R–R duration is measured in seconds but can be converted to degrees, where 360° represents the length of one cardiac cycle. Any areas of dyssynchronous contraction will appear as delays in the phase images and phase histogram.

In-house software was used to calculate synchrony, entropy, *ApEn*, *SampEn*, and phase SD from the RNVG phase images both pre and post beta-blockade. To calculate synchrony, each pixel within the ROI was defined by a vector where the length was defined as the amplitude (maximum change in counts), and the direction was defined as the phase angle. Synchrony is then defined as the vector sum of the pixel values divided by the scalar sum. A ventricle with completely synchronous contraction would have a synchrony value of 1 and a completely asynchronous contraction would have a synchrony value of 0. Entropy, as derived from Shannon information theory,^28^ was used as a measure of randomness in the phase histogram.^16^ A higher value of entropy indicates a more random contraction.

*ApEn* and *SampEn* were calculated using Eqs. 1 and 2, by considering the pixel values within the region of interest on the phase image as a data series. Both *ApEn* and *SampEn* calculate the probability that a series of pixels of length *m* from the phase image remains similar within a tolerance *r* at the next sequence in the data series. *SampEn* is a modification of *ApEn* described by Richman and Moorman,^24^ but unlike *ApEn*, *SampEn* displays relative consistency regardless of sequence length and tolerance values used, and it is independent of data length. *ApEn* is defined as

1 $$\begin{aligned} ApEn = - (N - m)^{-1} \sum _{i=1}^{N-m} \ln \left( \frac{C_{i^{m+1}} (r)}{C_{i^m}} \right), \end{aligned}$$

where *N* is the length of data, *m* is the sequence length, and *r* is the tolerance. $C{_{i^{m}}}(r)$ is the conditional probability that when a sequence of pixels is within the tolerance, then the next sequence will also be within tolerance. *SampEn* is defined as

2 $$\begin{aligned} SampEn = -ln \left( \frac{B^{m+1}(r)}{B^m(r)} \right) , \end{aligned}$$

3 $$\begin{aligned} B^m(r) = (N - m)^{-1}\sum _{i=1}^{N-m}B_i^m(r), \end{aligned}$$

where $B_i^m(r)$ is the number of sequences of length *m* that are within tolerance *r*, excluding self matches, and $B^m(r)$ is the probability that two sequences of length *m* are similar.

The values of input parameters *m* and *r* significantly affect the results so they must be optimized for the application. Based on the previous optimization work, *ApEn* was calculated using sequence length *m* = 2 and tolerance *r* = 7 and *SampEn* was calculated using sequence length *m* = 2 and tolerance *r* = 4^23^. To calculate *ApEn* and *SampEn*, each group of *m* = 2 pixels within the region of interest in the phase image was compared to every other group of *m* = 2 pixels within the region of interest. If they were similar within the defined tolerance *r*, it was counted as a match. This was carried out for every group of ‘*m*’ pixels then repeated with groups of ‘$m+1$.’ Unlike synchrony and entropy, *ApEn* and *SampEn* take into account the similarity of adjacent pixel values. A higher value of *ApEn* and *SampEn* would indicate a more dyssynchronous contraction. Correlation with LVEF and intra-operator variability was assessed for these novel dyssynchrony parameters.

### Statistical analysis

All data analysis and statistics were performed in R 3.6.3.^29,30^ Shapiro–Wilk’s test was used to check the normality for each parameter, and significance testing was performed, using the t-test or Wilcoxon rank-sum test, depending on the outcome of the univariate test of normality. For paired data, a paired two-sample *t* test or Wilcoxon signed-rank test was used. The chi-squared test was used to test the significance of categorical parameters. The correlation between the parameters was tested using Pearson’s correlation. A *P* value of < .05 was considered significant for all tests.

## Results

At baseline, there was no significant difference in sex, NYHA class, presence of hypertension, diabetes or ICD between the ischemic and non-ischemic groups. However, there was a significant difference in age (*P* < .001) and baseline heart rate (*P* = .02) when comparing the ischemic (age = 69 ± 9, HR = 82 ± 14) and non-ischemic (age = 54 ± 16, HR = 88 ± 16) patients. The correlation coefficients between LVEF and each dyssynchrony parameter were calculated to be .538 (*P* < .001) for synchrony, − .780 (*P* < .001) for entropy, − .338 (*P* = .001) for *ApEn* and − .675 for *SampEn* (*P* < .001) and − .602 (*P* < .001) for phase SD.

Intra-operator variability results are shown in Table 2. All of the parameters tested had excellent intra-operator variability with correlation coefficients ranging from .991 to .997. The original data were not available to test inter-operator variability.

**Table 2:** Intra-operator reproducibility

	ROI1	ROI2	Wilcoxon signed-rank	Pearson correlation
	Mean (± SD)	Mean (± SD)		
Synchrony	.90 (± .14)	.90 (± .13)	*P* = .945	.997 (*P* < .001)
Entropy	.70 (± .07)	.70 (± .07)	*P* = .994	.995 (*P* < .001)
ApEn	.54 (± .13)	.55 (± .31)	*P* = .894	.991 (*P* < .001)
SampEn	.91 (± .30)	.92 (± .30)	*P* = .967	.997 (*P* < .001)
Phase SD	28.7 (± 23.9)	28.6 (± 23.1)	*P* = .988	.997 (*P* < .001)

Comparison was made pre and post beta-blockade as summarized in Table 3. There was a significant improvement in all of the dyssynchrony parameters and LVEF measured post beta-blockade. The only parameter that did not significantly improve after beta-blockade was *ApEn* for the non-ischemic group. There was no significant difference in dyssynchrony between the ischemic and non-ischemic groups at baseline (*P* > .05). There was a weak relationship (correlation = .31, *P* = .004) between change in heart rate and change in LVEF post beta-blockade.

**Table 3:** Comparing parameters pre and post beta-blockade

	All			Ischemic (N = 54)			Non-ischemic (N = 32)		
	Pre BB	Post BB	*P* value	Pre BB	Post BB	*P* value	Pre BB	Post BB	*P* value
	Mean (± SD)	Mean (± SD)		Mean (± SD)	Mean (± SD)		Mean (± SD)	Mean (± SD)	
Synchrony	.90 (± 14 )	.95 (± .09 )	< .001	.91 (± .11)	.94 (± .09)	< .001	.87 (± .17)	.95 (± .08)	.001
Entropy	.70 (± .07 )	.65 (± .06 )	< .001	.70 (± .06)	.66 (± .07)	<.001	.70 (± .08)	.64 (± .06)	<.001
ApEn	.54 (± .13 )	.48 (± .13 )	< .001	.55 (± .12)	.47 (± .13)	.001	.54 (± .14)	.49 (± .14)	.103
SampEn	.91 (± .3 )	.72 (± .21 )	< .001	.86 (± .24)	.71 (± .2)	<.001	1.00 (± .37)	.74 (± .23)	.001
Phase SD	28 (± 21.2 )	18.8 (± 14.3 )	< .001	25.1 (± 16.8)	19.4 (± 14.5)	<.001	33 (± 26.6)	17.9 (± 14.1)	.001
LVEF (%)	21 (± 9 )	30 (± 11 )	< .001	21 (± 8)	28 (± 10)	<.001	20 (± 11)	32 (± 11)	<.001
HR	84 (± 15)	65 (± 14)	<.001	82 (± 14)	64 (± 15)	<.001	88 (± 16)	66 (± 14)	<.001

A paired significance test for each parameter was selected based on normality of the specific variable. An increase in synchrony, *ApEn* and *SampEn* indicate improved dyssynchrony, while a decrease in entropy or phase SD represent improved dyssynchrony. There was no significant difference in baseline dyssynchrony between the ischemic and non-ischemic groups

## Discussion

The results suggest that beta-blockers improve dyssynchrony for HFrEF of both ischemic and non-ischemic etiology. As expected LVEF also improves post beta-blockade. The results are consistent with the studies by Kaya et al and Takemoto et al.^15,31^ Both studies used septal to lateral delay as measured by echo to assess dyssynchrony and did not include any patients with ischemic heart failure. Kaya et al. found that beta-blockade improved LV synchrony and LVEF for heart failure patients with idiopathic dilated cardiomyopathy and LV dyssynchrony. Taketmoto et al. also found that patients with a QRS <120ms and sinus rhythm experienced an improvement in both LVEF and dyssynchrony after beta-blocker therapy. The mechanism for this improvement is not fully understood. Improved dyssynchrony has been linked with improved survival, as shown in CRT studies. For example, a sub-study of the EchoCRT trial found that persistent or worsening dyssynchrony six months post CRT was associated with worse clinical outcomes, in particular, heart failure hospitalizations.^32^

While the effect of dyssynchrony and beta-blockers on survival would be of clinical interest, this cohort is too small to provide any meaningful results. Treatment for these patients would have varied, and some went on to have PCI, CABG, or heart transplant after the study. Previous attempts to create models to predict mortality for heart failure patients have had only moderate accuracy, and those trying to predict a combined endpoint of hospitalization or death had even poorer results.^33,34^

There are currently no studies using newer echo dyssynchrony parameters, such as global longitudinal strain, to investigate the effect of beta-blockers on cardiac dyssynchrony. This study is the only work to date assessing dyssynchrony for heart failure patients using RNVG phase parameters. Further work to investigate the inter-operator variability should also be carried out, but good intra and inter-operator variabilities have been previously demonstrated for synchrony, entropy, and phase standard deviation.^16,18,21,35^ Heart failure treatment may benefit from further investigation of mechanical dyssynchrony in larger trials.

### Limitations

The results are from a single-center study, limited by a small patient sample with no control group. This study also assumes patient compliance with taking prescribed drugs. The authors also acknowledge the complexity in defining heart failure etiology. Some of the patients in the non-ischemic group may have mild ischemia.

## Conclusion

An improvement in dyssynchrony and LVEF was measured six months post beta-blockade for both ischemic and non-ischemic heart failure patients using novel phase parameters. A larger study with data from multiple centers would be desirable to confirm the results of this study.

## New Knowledge Gained

A significant improvement in dyssynchrony after beta-blockade therapy has been demonstrated for heart failure patients using novel measures of dyssynchrony from RNVG phase images.

## Abbreviations

LVEF: Left ventricular ejection fraction
RNVG: Radionuclide ventriculography
HF: Heart failure
HFrEF: Heart failure with reduced ejection fraction
MI: Myocardial Infarction
PCI: Percutaneous coronary intervention
CABG: Coronary artery bypass graft
COPD: Chronic obstructive pulmonary disease
PFTs: Pulmonary function tests
ICD: Implantable cardioverter defibrillator
HR: Heart rate
ApEn: Approximate entropy
SampEn: Sample entropy
SD: Standard deviation
NYHA: New York Heart Association
ESC: European Society of Cardiology
